# Type II Cochlear Ganglion Neurons Do Not Drive the Olivocochlear Reflex: Re-Examination of the Cochlear Phenotype in Peripherin Knock-Out Mice

**DOI:** 10.1523/ENEURO.0207-16.2016

**Published:** 2016-08-17

**Authors:** Stéphane Maison, Leslie D. Liberman, M. Charles Liberman

**Affiliations:** 1 Department of Otology and Laryngology, Harvard Medical School, Boston, Massachusetts 02115; 2 Eaton-Peabody Laboratories, Massachusetts Eye & Ear Infirmary, Boston, Massachusetts 02114-3096

**Keywords:** olivocochlear reflex, feedback, peripherin, hair cells, cochlea

## Abstract

The cochlear nerve includes a small population of unmyelinated sensory fibers connecting outer hair cells to the brain. The functional role of these type II afferent neurons is controversial, because neurophysiological data are sparse. A recent study (Froud et al., 2015) reported that targeted deletion of peripherin, a type of neurofilament, eliminated type II afferents and inactivated efferent feedback to the outer hair cells, thereby suggesting that type II afferents were the sensory drive to this sound-evoked, negative-feedback reflex, the olivocochlear pathway. Here, we re-evaluated the cochlear phenotype in mice from the peripherin knock-out line and show that (1) type II afferent terminals are present in normal number and (2) olivocochlear suppression of cochlear responses is absent even when this efferent pathway is directly activated by shocks. We conclude that type II neurons are not the sensory drive for the efferent reflex and that peripherin deletion likely causes dysfunction of synaptic transmission between olivocochlear terminals and their peripheral targets.

## Significance Statement

Recent studies present the following opposing views on the role of unmyelinated sensory fibers in the auditory nerve: one suggests that type II spiral ganglion neurons are nociceptors mediating auditory pain (Flores et al., 2015); and another suggests that they comprise the sensory limb of the cochlear efferent reflex (Froud et al., 2015). Both cannot be correct, since nociceptors respond at traumatically high sound levels, whereas cochlear efferents respond near the hearing threshold. Here, we re-examine the phenotype of the mutant mouse on which the latter case was based: we show that the type II innervation is not missing in this mutant, as claimed, and that the loss of efferent feedback is due to a defect in efferent transmission, rather than a loss of sensory drive.

## Introduction

The primary sensory fibers projecting from cochlear hair cells to the cochlear nucleus are of the following two fundamentally different neuronal types: myelinated, type I neurons innervating inner hair cells; and unmyelinated type II neurons innervating outer hair cells (Spoendlin, 1969). Because type I neurons outnumber type II neurons by 20:1 (Spoendlin, 1969), and because the axons of type II neurons are too small to be sampled by conventional micropipette recordings (Kiang et al., 1982; Liberman, 1982), the sound-evoked and spontaneous discharge patterns of type I neurons are extremely well characterized (Liberman, 1978), whereas the neurophysiology and, indeed, the functional significance of the type II population have remained enigmatic (Brown, 1994; Robertson et al., 1999).

By analogy to the somatosensory system, it has been suggested that the unmyelinated type II fiber pathway might mediate the sensation of auditory pain (Simmons and Liberman, 1988). Indeed, the few successful recordings from type II neurons *in vivo* have suggested that they do not respond to sound at sound pressure levels up to 90 dB above the thresholds in type I cochlear neurons (Robertson, 1984; Brown, 1994) that are comparable to the threshold of hearing (Kiang et al., 1965). Recent research, both *in vitro* and *in vivo*, has lent support to the notion that the type II fibers are nociceptors (Flores et al., 2015; Liu et al., 2015), responding only when there is damage to the outer hair cells (OHCs).

In contrast to this idea, a recent report (Froud et al., 2015) suggested that type II neurons are the sensory drive to the medial olivocochlear (MOC) neurons, a negative feedback neuronal circuit projecting from the brainstem to the outer hair cells. The MOC reflex, when activated, raises cochlear thresholds by suppressing the electromotility of OHCs and thereby turning down the gain of the “cochlear amplifier” (for review, see Guinan, 2010). Since MOC neurons have thresholds within 10-20 dB of type I thresholds (Liberman and Brown, 1986; Liberman, 1988a,b; Brown et al., 1998), type II neurons would have to be similarly sensitive to sound if they represented the sensory limb of this feedback arc. The evidence for this new hypothesis was derived from a mouse with targeted deletion of the gene for peripherin (Prph), an intermediate filament expressed mainly in the neurons of the peripheral nervous system (Lee and Cleveland, 1996) and, notably, strongly expressed in the cell bodies of type II (and not type I) cochlear spiral ganglion cells (Hafidi, 1998). The study reported that the sound-evoked MOC reflex was greatly attenuated in Prph^−/−^ ears compared with Prph^+/+^ ears, and that the type II innervation of the cochlea was absent in the knockout (KO; Froud et al., 2015).

Here, we have re-evaluated the phenotype of the same Prph^−/−^ line studied previously (Larivière et al., 2002). In contrast to the prior study, we find that the type II innervation of OHCs is essentially unaltered in the peripherin knockouts. We also show that peripherin is expressed in some of the MOC neurons themselves, as well as in type II cell bodies. By electrically activating the MOC bundle, we show that the reflex inactivation observed previously can be explained by the loss of MOC function per se (i.e., the cochlear suppression that is normally evoked by shocking the MOC bundle is absent in the knockout). Thus, there is no basis for concluding that type II neurons provide the sensory drive to the MOC reflex.

## Materials and Methods

### Animals and groups

Mice heterozygous for the targeted deletion of the peripherin gene were obtained from their laboratory of origin (Larivière et al., 2002), as were the mice from the prior cochlear study that inspired the present re-evaluation (Froud et al., 2015). Mice were bred in our own animal care facility and genotyped by Transnetyx using the lacZ insert in the knock-out line. As in the prior study, homozygous knockouts and wild types (WTs) of either sex were identified for histological and physiological study from 6.5 to 8.5 weeks of age. All studies were approved by the institutional animal care and use committee at the Massachusetts Eye and Ear Infirmary.

### Cochlear function tests

Auditory brainstem responses (ABRs) and distortion product otoacoustic emissions (DPOAEs) were recorded while mice were anesthetized with ketamine and xylazine. ABR stimuli were 5 ms tone pips with a 0.5 ms rise–fall time delivered at 30/s. Sound level was raised in increments of 5 dB, from 10 dB below threshold to 90 dB SPL. The threshold for ABR was defined as the lowest stimulus level at which a repeatable waveform morphology could be identified in the response. DPOAEs were recorded for primary tones with a frequency ratio of 1.2 and with the level of the *f*_2_ primary 10 dB less than the *f*_1_ level, combined in increments of 5 dB. The 2*f*_1_–*f*_2_ DPOAE amplitude and surrounding noise floor were extracted. The threshold for DPOAEs is defined as the *f*_1_ level required to produce a response amplitude of 0 dB SPL.

### Olivocochlear function tests

After anesthetization with urethane (1.20 g/kg, i.p.) and xylazine (20 mg/kg, i.p.), a posterior craniotomy and partial cerebellar aspiration exposed the floor of the fourth ventricle. To stimulate the olivocochlear bundle, shocks (monophasic pulses, 150 μs duration, 200/s) were applied through fine silver wires (0.4 mm spacing) placed along the midline, spanning the olivocochlear decussation. The shock threshold for facial twitches was determined, muscle paralysis was induced with α-d-tubocurarine (1.25 mg/kg, i.p.), and the animal was connected to a respirator via a tracheal cannula. Shock levels were raised to 6 dB above twitch threshold. During the olivocochlear suppression assay, the *f*_2_ level was set to produce a DPOAE 10–15 dB above the noise floor. To measure the olivocochlear effects, repeated measures of baseline DPOAE amplitude were first obtained (*n* = 25), followed by a series of 70 contiguous periods in which DPOAE amplitudes were measured with simultaneous shocks to the olivocochlear bundle and additional periods during which DPOAE measures continued after the termination of the shock train.

### Histological preparation

Animals were anesthetized with ketamine and perfused intracardially with 4% paraformaldehyde in PBS at pH 7.3. Immediately afterward, fix was flushed through the cochlear scalae; the cochleae were then extracted and postfixed for 2 h at room temperature. Cochleae were transferred into 0.12 m EDTA and decalcified for 2 d at room temperature. Each cochlea was then dissected into six pieces (approximately half turns of the cochlear spiral) for whole-mount processing of the cochlear epithelium. Pieces were permeabilized with a freeze/thaw cycle, as follows: cryoprotected in 30% sucrose for 15 min, frozen on dry ice, thawed, and rinsed in PBS for 15 min. Immunostaining began with a blocking buffer (PBS with 5% normal horse serum and 0.3% Triton X-100) for 1 h at room temperature and was followed by overnight incubation at 37ºC with some combination of the following primary antibodies: (1) rabbit anti-peripherin (catalog #ab4666, Abcam) at 1:200; (2) goat anti-Na^+^/K^+^-ATPase α3 (C-16; catalog #sc-16052, Santa Cruz Biotechnology) at 1:200 to label type I afferents and MOC efferents; (3) chicken anti-NF-H (neurofilament; catalog #AB5539, Chemicon) at 1:1000, or mouse anti-NF200 (catalog #69705, MP Biomedicals) at 1:50,000, or mouse anti-TuJ1 (β-tubulin III; catalog #MMS-435P, Covance) at 1:2000 to label cochlear afferent and efferent fibers; (4) goat anti-parvalbumin (catalog #PVG-214, Swant) at 1:2000, to delineate type II outer spiral fibers and their terminal swellings; (5) mouse anti-synaptophysin (catalog #69730, MP Biomedicals) at 1:100, or rabbit anti-VAT [vesicular acetylcholine (ACh) transporter; catalog #ab68986, Abcam] at 1:200, to label terminals of cochlear efferent fibers; (6) mouse anti-CtBP2 (C-terminal binding protein; catalog #612044, BD Biosciences) at 1:200, to quantify presynaptic ribbons; and/or (7) rabbit anti-myosin VIIa (catalog #25-6790, Proteus Biosciences) at 1:200 to delineate the hair cell cytoplasm. Primary incubations were followed by two sequential 60 min incubations at 37°C in species-appropriate secondary antibodies (coupled to Alexa Fluor dyes) with 0.3% Triton-X. After immunostaining and mounting of dissected pieces in Vectashield, slides were coverslipped and sealed with nail polish.

### Cochlear frequency mapping

After immunostaining, each cochlea was mapped in ImageJ using a spline fit to a set of user-positioned points placed along the arc of the pillar heads in a photomicrograph of each dissected piece. A custom plugin to ImageJ computes the cumulative length, and displays the positions of designated half-octave frequency points (5.6, 8.0, 11.3, 16.0, 22.6, 32.0, 45.2, and 64 kHz) in each case, as determined by the cochlear frequency map for the mouse (Müller et al., 2005). Printouts of the maps for each case provide a “roadmap” to guide acquisition of images at precisely stereotyped positions in all cases.

### Image acquisition

At each of the eight half-octave frequency points along the cochlear spiral, *z*-stacks were acquired using a 63× glycerol-immersion objective (numerical aperture, 1.3) on a Leica SP8 confocal microscope, a raster size of 1024 × 512 and a resultant pixel size of 75 nm in the *x–y*-plane and a *z*-*s*tep of 0.33 μm between optical slices. The *z*-span was always carefully adjusted to include all synaptic elements in all the hair cells in the stack. Laser power, acquisition filters, and photomultiplier tube (PMT) gains were always carefully adjusted to minimize pixel saturation in all channels, to maximize the full use of the dynamic range, and to eliminate interchannel crossover in the acquired signals; however, alterations in acquisition parameters were minimal within or across cases. At the standard magnification and zoom, each stack spanned ∼77 μm of cochlea length (i.e., about 9 adjacent IHCs and 10 adjacent OHCs in each of the three rows). In the OHC area, two adjacent sets of OHCs were always imaged at each of the designated frequency regions in each ear.

### Morphometric analysis

Morphometric analysis in the present study included (1) counts of OHC ribbons and (2) estimation of the density of efferent innervation in the OHC area. Cases to be quantified were immunostained together and imaged together, using exactly the same laser power and PMT gains for all image stacks.

#### Ribbon/synapse counts

The signal-to-noise ratio in the “ribbon channel” is high enough that the identification of CtBP2-positive puncta is achievable by computer algorithm without any user adjustment. Each acquired *z*-stack is ported into Amira software, where the “connected components” function is used to identify the *x*-, *y*-, *z*-positions and volumes of every element in 3-D voxel space of at least 10 contiguous pixels and within which all the pixel intensities are >40 on an 8 bit (0–255) scale. The total number of ribbons is then divided by the number of hair cells in the stack (including fractions), as assessed using either the Myosin VIIa channel or the faint cytoplasmic labeling in the parvalbumin channel in the native *z*-stack.

#### Efferent innervation density

To assess the density of efferent innervation in each *z*-stack, the channel containing the fluorescence signal from the anti-VAT immunolabeling is extracted from the confocal image file, and a maximum projection obtained in the *x–y*-plane and exported as a one-color image file. This image file is ported to ImageJ, where a thresholding algorithm is applied and the total silhouette area of the suprathreshold pixels is computed. An intensity value of 45 on an 8-bit (0–255) scale was used as threshold for all images in all cases.

### Statistical analysis

Unless otherwise indicated, all statistical analysis was performed using two-way ANOVA; *p* values <0.01 for any intergroup differences were considered to be significant.

## Results

### Immunostaining for type II fibers and MOC efferents

**Figure 1.**
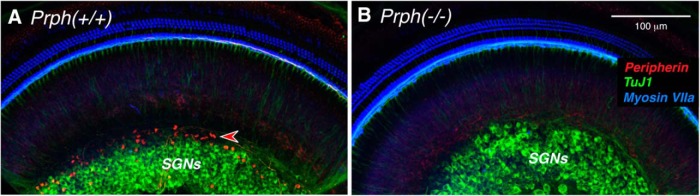
***A***, ***B***, Anti-peripherin (red) immunostains the cell bodies of type II SGNs in Prph^+/+^ ears (***A***) but not in Prph^−/−^ (***B***), confirming the null expression in the knockout. Each image is a maximum projection of a confocal *z*-stack through the apical turn. Antibodies to β-tubulin (TuJ1; green) stain the cell bodies of type I SGNs, and myosin VIIa is used to stain hair cells (blue). Scale bar: ***B*** (for ***A***, ***B***), 100 μm.

We obtained mice heterozygous for targeted deletion of the peripherin gene (Larivière et al., 2002) and bred Prph^−/−^ and Prph^+/+^ mice for the present study. To validate the gene deletion in our animals, we first showed that peripherin expression was indeed eliminated. Immunostaining in WT cochleae shows a subset of spiral ganglion neurons (SGNs) strongly immunopositive for peripherin (Fig. 1*A*, red), while the great majority are labeled with antibodies to β-tubulin (Fig. 1*A*, TuJ1-green). Consistent with prior reports, the peripherin-positive type II neurons constitute a minority of the SGN population and tend to be located toward the periphery of the ganglion (Huang et al., 2007). As expected, the peripherin immunoreactivity is missing in the KO ears. One example is shown in Figure 1*B*. To double check our genotyping results, we immunostained four pairs of KO/WT ears with peripherin. Type II SGNs were brightly stained in all four WTs, and peripherin staining was absent in all four KOs.

**Figure 2.**
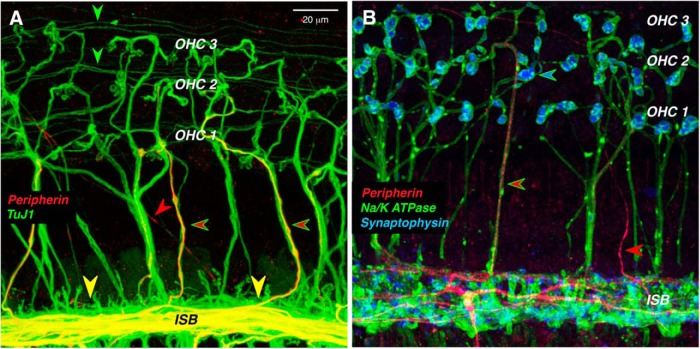
In the organ of Corti, peripherin weakly stains the peripheral projections of type II SGNs, while it strongly stains a subset of MOC efferents. ***A***, Thin outer spiral fibers (green arrowheads) in the outer spiral bundles are immunopositive for β-tubulin (TuJ1), as are thick MOC fibers running radially across the tunnel of Corti. Some of MOC fibers are also positive for peripherin (green-rimmed red arrowheads). Peripherin also strongly stains MOC fibers in the inner spiral bundle (ISB) and, rarely, a thin type II projection running diagonally across the floor of the tunnel (red arrowhead). ***B***, Antibodies to synaptophysin (blue) and Na^+^/K^+^ ATPase (green) identify the thick tunnel-crossing fibers as MOC neurons, a subset of which are peripherin positive (green-rimmed red arrowhead). An occasional thin type II projection is also peripherin positive (red arrowhead). Images are maximum projections from the 11.3 kHz region of two different wild-type animals. Scale bar: ***A*** (for ***A***, ***B***), 20 μm.

**Figure 3.**
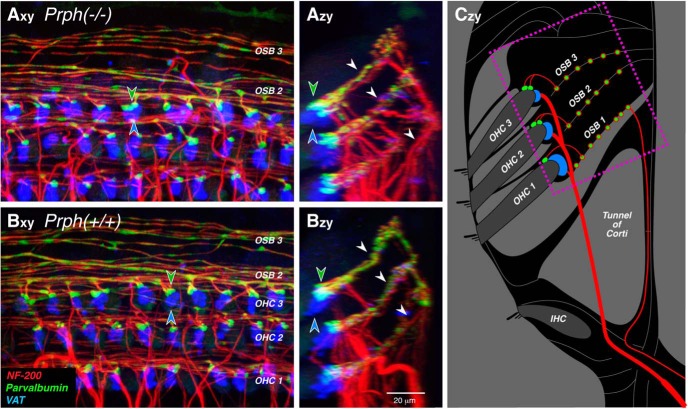
Immunostaining for parvalbumin (which stains type II terminals and outer spiral fibers green) and vesicular acetylcholine transporter (which stains MOC terminals blue) suggests that both afferent and efferent innervations are normal in Prph^−/−^ ears. ***A***, ***B***, Maximum projections of confocal *z*-stacks from the 16 kHz region of a Prph^−/−^ ear (***A***) and a Prph^+/+^ ear (***B***), shown in the acquisition plane (*x–y*) and in the orthogonal plane (*z–y*) showing a cross-sectional view, as schematized in ***C***. The dotted box in ***C*** shows the approximate region imaged in ***A*** and ***B***. Green-filled and blue-filled arrowheads in ***A*** and ***B*** highlight the spatially offset clusters of type II and olivocochlear terminals, respectively, underneath the third-row OHCs. White arrowheads in ***Azy*** and ***Bzy*** point to the three outer spiral bundles (OSBs) running between the Deiter’s cells. Scale bar: ***Bzy*** (for all panels), 20 μm.

Although peripherin is a robust marker of type II cell bodies in the spiral ganglion, it weakly and infrequently labels type II peripheral projections in the adult organ of Corti (Fig. 2, red arrowheads). Each type II SGN sends an unmyelinated peripheral axon from the ganglion to the sensory epithelium, where it crosses along the floor of the tunnel of Corti to the OHC area (Fig. 3*C*), turns toward the base of the cochlea, and spirals in the outer spiral bundles between adjacent Deiter’s cells for up to a millimeter, before branching to innervate as many as 100 OHCs (Brown, 1987a; Simmons and Liberman, 1988). The thin, spiraling, type II projections in the outer spiral bundles are immunopositive for TuJ1 (Fig. 2*A*, green arrowheads), as are the radially directed, and thicker, axons of the MOC efferents (Fig 2*A*, green-rimmed red arrowheads). However, of the two fiber types in the OHC area, only the MOC axons are immunopositive for a Na^+^/K^+^ ATPase (Fig. 2*B*; McLean et al., 2009). Although peripherin is a weak marker of the outer spiral bundles, it robustly labels a subset of the MOC efferents as they spiral in the inner spiral bundle, and as they cross the tunnel of Corti (Fig. 2*A*,*B*). Prior single-fiber labeling studies show that type II fibers never spiral in the inner spiral bundle, whereas most MOC fibers do, before crossing to the OHC region (Brown, 1987a,b; Simmons and Liberman, 1988). Prior studies showing robust peripherin immunolabeling of spiraling type II projections in the outer spiral bundles have all been in neonatal ears (Barclay et al., 2011).

To immunostain the synaptic terminals of type II and MOC efferents, we used antibodies to parvalbumin and VAT (Maison et al., 2003), respectively (Fig. 3). In both WT (Fig. 3*B*) and KO (Fig. 3*A*) ears, there is at least one type II terminal and MOC efferent terminal on almost every OHC from each of the three rows, and there is no obvious difference in size or number of either terminal type between the two genotypes. As reported previously using a reporter mouse for GABA_B_ receptors (Maison et al., 2009), type II terminals tend to be found on the distal sides of the OHCs. This trend is clear in both the *x–y* and *y–z* projections in both WT and KO ears (Fig. 3). Parvalbumin also immunostains the thin, type II preterminal fibers spiraling in the outer spiral bundles under the OHCs (Fig. 3), as do antibodies to a 200 kDa neurofilament protein (Fig. 3; NF-200, red). The images in Figure 3 suggest no difference in the numbers of type II spiraling fibers between the two genotypes.

**Figure 4.**
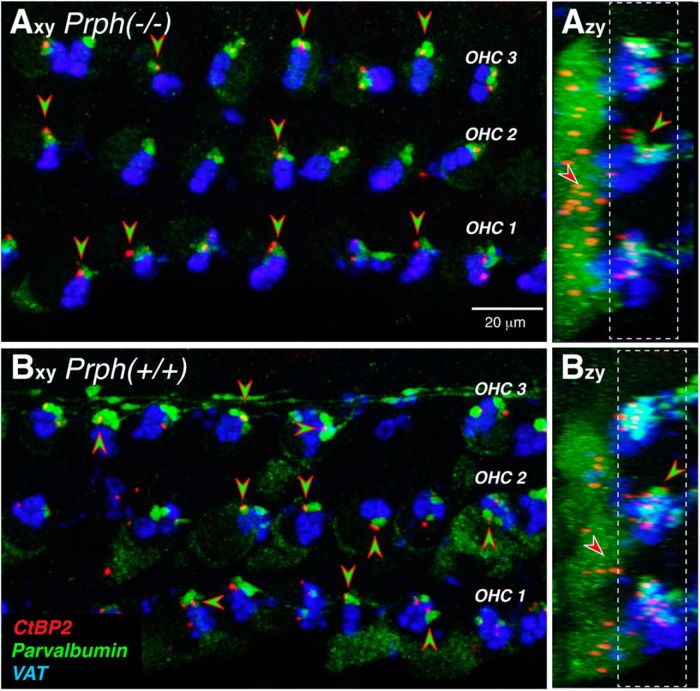
Type II terminals are apposed to presynaptic ribbons in Prph^−/−^ and Prph^+/+^ ears. ***A***, ***B***, Maximum projections of confocal *z*-stacks from the 11.3 kHz region of a knock-out and a wild-type ear, shown in the acquisition plane (*x–y*) and the orthogonal plane (*z–y*; Fig. 3*C*). Green-filled, red-rimmed arrowheads point to appositions between (parvalbumin-positive) type II terminals and (CtBP2-positive) presynaptic ribbons. Red-filled, white-rimmed arrowheads in the *z–y* projections point to nonsynaptic ribbons. Scale bar and immunostaining keys apply to all panels. Dashed white boxes in the *z–y* projections show the *z*-cropping used to generate the *x–y* projections.

To definitively identify the parvalbumin-positive boutons as type II terminals, we added antibodies to CtBP2, a major protein in the presynaptic ribbons (Schmitz, 2009) seen at both type I and type II synapses (Hashimoto and Kimura, 1988; Hashimoto et al., 1990; Liberman et al., 1990). As can be seen in Figure 4, the ribbon puncta are clearly paired with the parvalbumin-positive boutons in both WT and KO ears, as expected for markers of type II afferent synapses.

**Figure 5.**
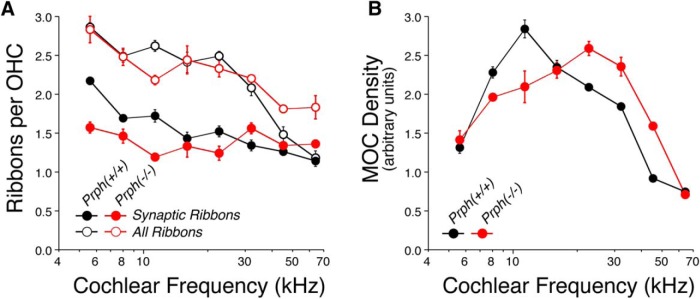
***A***, ***B***, Quantitative analysis shows that the afferent (***A***) and efferent (***B***) innervation of OHCs is similar in Prph^−/−^ and Prph^+/+^ ears. ***A***, Mean ribbon counts and synaptic ribbon counts per OHC (±SEM) as a function of cochlear location. ***B***, Mean silhouette area of MOC terminals per OHC (±SEM) as a function of cochlear location. Both graphs are based on data from four cochleae, two of each genotype. Each point is based on data from four high-power *z*-stacks, each containing ∼25 OHCs.

To quantitatively assess the effects of peripherin deletion on the density of afferent and efferent innervation in the OHC area, we batch processed and batch imaged two ears of each genotype, immunostained as shown in Figure 4. Our ribbon counts at eight equally spaced cochlear locations from the apical to the basal extreme of the cochlear spiral suggest that the type II innervation is essentially unchanged in the KO ears (Fig. 5*A*). As reported previously, and as shown in the *z–y* projections of Figure 4, the CtBP2-positive puncta in each OHC are seen in the following two clusters: one at the basal pole of each OHC, where the afferent terminals are found; and a second set of “nonsynaptic” ribbons located in the circumnuclear zone (Maison et al., 2016). Our counts (Fig. 5*A*) show no difference between genotypes in either the synaptic ribbon counts or the total ribbon counts: the group effects were not significant, as determined by two-way ANOVA (*p* = 0.107, *F* = 7.863, and *p* = 1.0, *F* = 0, respectively). We also measured the silhouette areas of MOC terminals in the same sets of *z*-stacks and again saw no significant difference between genotypes (Fig. 5*B*; *p* = 0.412, *F* = 1.055). It may be significant that, for both synaptic counts and MOC innervation density, there is a slight decrease in KO ears compared with WT ears in the cochlear apex, but a slight increase in KO ears compared with WT ears in the base. The qualitative trends captured in these four batch-processed ears were verified by qualitative evaluation of four additional ears of each genotype, immunostained for parvalbumin and an MOC terminal marker [either VAT (Maison et al., 2003) or synaptophysin (Counter et al., 1997)], and imaged at the same eight locations in each ear.

### Evaluating cochlear function and MOC-mediated suppression

Routine evaluation of cochlear function in experimental animals can be accomplished by two minimally invasive measures: ABRs and DPOAEs. ABRs represent the synchronized, summed activity of cochlear type I neurons recorded from needle electrodes in the scalp in response to transient tone-pip stimuli. DPOAEs are created in the cochlea as distortions in the hair cell transduction process in response to two continuous tones (*f*_1_ and *f*_2_), which are reverse transduced into mechanical distortions by OHC electromotility, amplified, and back-propagated as pressure waves through the middle ear bones, where they radiate into the external ear as changes in ear canal sound pressure.

**Figure 6.**
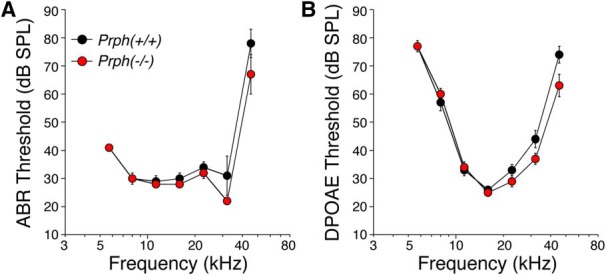
Cochlear thresholds are similar in Prph^−/−^ and Prph^+/+^ ears. Data are the means (±SEMs) from eight ears of either genotype.

We measured thresholds for both ABRs and DPOAEs in eight mice of each genotype at eight log-spaced frequencies, corresponding to the eight cochlear locations where we captured confocal images of the organ of Corti. As seen in Figure 6, the mean thresholds in the two groups were very similar. Although there is a trend toward better thresholds in the Prph^−/−^ ears than in the Prph^+/+^ ears, these small differences were not significant by two-way ANOVA (DPOAEs, *p* = 0.125, *F* = 2.5; ABRs, *p* = 0.106, *F* = 3.017).

**Figure 7.**
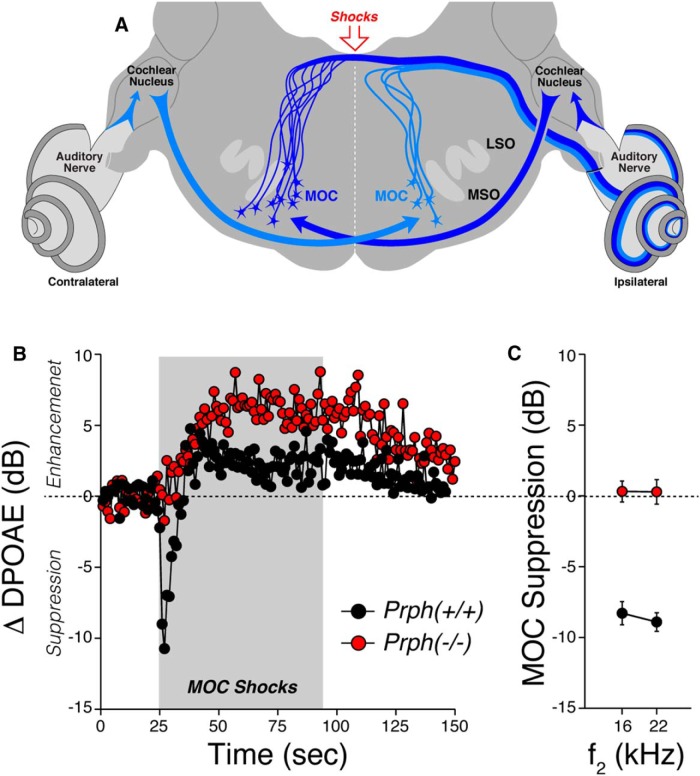
Shock-evoked MOC suppression is absent in Prph^−/−^ ears. ***A***, Schematic cross-section through the brainstem at the level of the lateral superior olive (LSO) and medial superior olive (MSO), showing (1) the cell bodies of MOC neurons projecting to one ear (ipsilateral), (2) the circuit underlying the sound-evoked MOC reflex, and (3) the location of the electrical stimulator at the floor of the fourth ventricle. ***B***, Mean time course of the changes in DPOAE amplitude (compared with mean preshock baseline) evoked by a 70 s shock train to the OC bundle. Data are averaged from four WT and two KO ears. DPOAEs in the ipsilateral ear were evoked with primary tones at 16.0 kHz (*f*_2_) and 13.33 kHz (*f*_1_), presented at levels evoking a DPOAE 10 dB above the noise floor. ***C***, Mean shock-evoked suppression in DPOAE amplitudes for *f*_2_ = 16 or 22.6 kHz for Prph^−/−^ vs. Prph^+/+^ ears (*n* = 2 and *n* = 4, respectively). To collapse traces such as those in ***B*** to a single number, we average, for each test iteration, the first three points after shock onset.

The function of the MOC efferent pathway can be assessed either by adding an elicitor sound (usually to the contralateral ear), or by directly shocking the efferent bundle with electrodes placed on the floor of the fourth ventricle, as schematized in Figure 7*A*. When activated by either means, MOC terminals release ACh, which binds to nicotinic ACh receptors on OHCs, increasing Ca^++^ influx and activating nearby K^+^ channels, which hyperpolarizes the OHCs and reduces their contribution to cochlear amplification (Elgoyhen et al., 2009). These MOC effects, whether sound or shock evoked, are easily quantified by monitoring the amplitude of DPOAEs in the ipsilateral ear before, during, and after the period of MOC activation (Puria et al., 1996). In the anesthetized mouse, the sound-evoked reflex is greatly attenuated (Chambers et al., 2012), and the ipsilateral suppression evoked by contralateral sound is complicated by intermixing of effects from the middle ear muscle reflex and other sources (Maison et al., 2012).

Thus, in the present study, we directly activated the MOC bundle by electrical stimulation at the floor of the fourth ventricle, while the mice were paralyzed to remove the effects of activating the middle ear muscles. In normal mice, activating the MOC with shocks causes a large (∼10 dB) and immediate suppression of the DPOAEs, followed by a slow return to baseline and “overshoot” of DPOAE amplitudes, despite continued shocking of the bundle (Fig. 7*B*). This complex behavior seen in Prph^+/+^ ears has been seen in other mouse studies of shock-evoked MOC effects (Maison et al., 2007). In contrast, the shock-evoked effects in the Prph^−/−^ ears show no fast suppression, only a slow enhancement of DPOAE amplitudes. This pattern is similar to that seen in mice with targeted deletion of either of the nicotinic ACh receptors (α9 or α10) or the calcium-activated K^+^ channels (SK or BK) expressed at these MOC synapses (Maison et al., 2013). The mechanisms underlying this slow MOC-mediated enhancement are not clear (Maison et al., 2007).

We tested MOC effects on DPOAEs evoked at *f*_2_ = 16 and 22.6 kHz (Fig. 7*C*) because the maximum shock-evoked effects are seen in this frequency region in mice (Maison et al., 2007). The differences between WT and KO mice are highly significant (*p* < 0.001) at both 16 and 22.6 kHz. The present results reveal that the efferent limb of the MOC reflex is nonfunctional, at least with respect to its normal suppressive effect. Thus, the loss of contralateral sound evoked suppression of DPOAEs reported in a prior study of these Prph^−/−^ mice need not be attributed to the loss of sensory drive, as previously suggested (Froud et al., 2015).

## Discussion

### Resolving the anatomical discrepancy

Peripherin is a type of “intermediate filament” [i.e., a class of structural proteins with diameters (8–10 nm) intermediate between actin (6 nm) and microtubules (24 nm; Lee and Cleveland, 1996)]. Although widely expressed during development, peripherin in the adult is mostly expressed by neurons of the peripheral nervous system and has long been known as a robust marker of type II SGNs (Hafidi, 1998), the small unmyelinated neurons in the cochlear sensory nerve that selectively innervate OHCs (Kiang et al., 1982). Mice with targeted deletion of the gene for peripherin show selective loss of a subset of the unmyelinated sensory nerves of the dorsal root ganglion, without any apparent loss of the larger myelinated sensory fibers (Larivière et al., 2002).

These observations inspired Froud et al. (2015) to investigate the cochlear phenotype in the Prph^−/−^ mouse, presumably in hopes of studying the peripheral auditory system in a mouse model with selective lack or dysfunction of the type II afferent pathway. They reported that cochleae in this knock-out line “lack type II SGN innervation of OHCs” and also lack contralateral sound suppression of DPOAEs. Based on these findings, they suggested that the “type II SGN sensory transmission drives the MOC efferent regulation of cochlear amplifier gain.”

Here, we conclude that the type II innervation of OHCs is essentially unchanged in mice from the same Prph^−/−^ line studied by the Housley laboratory. How could the two studies come to such different conclusions? We both used NF-200 immunostaining to label type II fibers in the outer spiral bundles under the OHCs. Comparison of our respective confocal images shows many more outer spiral fibers in our WT ears (Fig. 3*A*) than theirs (Froud et al., 2015, their Fig 1c). Given the extremely small caliber of type II projections (Fig. 2*A*), their immunostaining may not have been robust enough to detect all the outer spiral fibers in either WT or KO ears. It may also be relevant that the confocal projection that Froud et al. (2015) use to illustrate the KO phenotype, both as a maximum projection (Fig. 1*C*) and as a 3-D movie (Froud et al., 2015, their Supplementary Movie 2), shows tunnel-crossing axons ending blindly in the middle of the tunnel. Since cochlear axons do not end in this fashion, the *z*-stack must be incomplete (i.e., did not go “deep” enough into the sensory epithelium to capture the entire spans of the afferent and efferent fibers bundles in the sensory epithelium).

To further clarify the anatomical facts, we added parvalbumin and CtBP2 immunostaining to the analysis to label type II terminal boutons and presynaptic ribbons (Figs. 3, 4). With these markers, the mean synaptic counts from 50 OHCs from each genotype at each of eight locations along the cochlear spiral (for a total of ∼800 OHCs in our sample) provide compelling evidence for the essential integrity of the type II innervation in the Prph^−/−^ ears (Fig. 5).

The prior study also provided ultrastructural data from serial blockface reconstruction of approximately nine OHCs from each genotype and concluded that there were no afferent terminals on any of the KO OHCs (Froud et al., 2015, their Supplementary Fig. 2). In our confocal analysis of eight cochlear regions in each of six KO ears, we never saw nine adjacent OHCs without any parvalbumin-positive terminals or synaptic ribbons; thus, this discrepancy is hard to dismiss as a small-sample anomaly. However, the resolution of serial blockface ultrastructure used by Froud et al. (2015) is not as good as that offered by classic ultrathin sections, and distinguishing afferent from efferent terminals in the OHC area can be difficult even in transmission electron microscopy. This is especially true because, at least in cat and human (Nadol, 1981; Thiers et al., 2008), type II terminals make reciprocal synapses with the OHCs (i.e., each type II terminal makes both afferent and efferent synapses with the OHCs). Thus, it is possible that afferent terminals in the KO ears were misclassified as efferent in origin.

### Type II physiology and the MOC reflex

The prior study of Prph^−/−^ ears (Froud et al., 2015) reported that the strength of the MOC reflex was attenuated in the KO compared with the WT, based on measuring contralateral sound-evoked suppression of ipsilateral DPOAEs. Froud et al. (2015) suggested that this reflex attenuation arose because type II neurons normally provide the sensory drive to the MOC neuronal circuitry.

Of course, if type II neurons are not missing in the Prph^−/−^ ears, there is no basis for this speculation. Nevertheless, we show here that, in the Prph^−/−^ ears, the MOC neurons per se are dysfunctional when directly activated by electric shocks. Thus, the reflex attenuation in the Prph^−/−^ ears likely arises from dysfunction in the efferent, not the afferent, limb of this reflex arc. We also showed that peripherin is normally expressed in both type II neurons and some MOC efferent projections (Figs. 1, 2). Thus, the loss of MOC reflex function could arise from developmental anomalies in the MOC/OHC synapses due to the constitutive lack of peripherin, which is likely more widely expressed in both cochlear afferents and efferents during development (Lee and Cleveland, 1996; Hafidi, 1998). The shock-evoked MOC phenotype that we observed in the Prph^−/−^ ears is similar to that seen in the ears with targeted deletion of either of the nicotinic ACh receptors that mediate MOC synaptic transmission at the OHCs (Maison et al., 2007). As observed in the Prph^−/−^ ears, both α9 and α10 KO ears show a nearly normal complement of cholinergic terminals at the bases of OHCs, despite being functionally de-efferented, as demonstrated via the measurement of shock-evoked MOC suppression (Vetter et al., 1999, 2007).

The idea that type II afferent neurons might provide the sensory drive for the MOC reflex is problematic for other reasons. Neurophysiological studies of single MOC neurons show, in both cats and guinea pigs, that they respond to sound at intensities within 10–20 dB of type I neurons, and correspondingly within 10–20 dB of behavioral thresholds (Liberman, 1988a,b; Brown et al., 1998). On the other hand, existing recordings from the type II cell bodies, though few in number, suggest that these small unmyelinated neurons, comprising only 5% of the cochlear nerve, do not respond to sound up to intensities of 80–90 dB SPL (Robertson, 1984; Brown, 1994). Furthermore, consistent with their small caliber and lack of myelination, type II antidromic response latencies to brainstem shocks are 6–7 ms, compared with <1 ms for the larger, myelinated type I neurons (Brown, 1994). In contrast, the sound-evoked latencies of single MOC efferents can be as short as 4.5 ms, which is only 3.5 ms slower than the response of some type I afferents (Brown et al., 2003). Thus, it is difficult to suggest that type II afferents are the primary sensory drive of the MOC reflex loop without discounting much of the existing literature on type II and MOC neurophysiology.

By analogy to the role of the unmyelinated fibers in the somatosensory system, a long-standing hypothesis about the type II neurons of the cochlea is that they are nociceptors and mediate the sensation of auditory pain (Simmons and Liberman, 1988). It is interesting that, in the adult somatosensory system, peripherin is expressed in a subset of unmyelinated nociceptors (Larivière et al., 2002). A recent *in vivo* study used c-Fos activation in the cochlear nucleus to suggest that type II central projections may be activated only after sound exposures that damage OHCs (Flores et al., 2015), and an *in vitro* patch-clamp study of type II terminals in cochlear explants showed that glutamatergic responses are extremely weak and that robust responses are evoked only by ATP and/or by damaging nearby OHCs (Liu et al., 2015).

It remains an interesting idea that the type II system can modulate the activity of the MOC circuitry, although there is no direct evidence for it. Many studies have shown that an intact MOC reflex minimizes noise-induced threshold elevation and OHC damage (Rajan, 1991), and it would be a useful design feature for incipient OHC damage to increase the gain of this negative feedback system. Indeed, prior neurophysiological studies in the cat have shown that 10 min presentations of high-level noise at near-traumatic levels (>90 dB SPL) can elicit long-lasting enhancements of the sound-evoked discharge rates in single MOC neurons (Liberman, 1988b). Such effects could be protective and could conceivably represent interactions between the afferent and efferent innervation in the OHC area.

## Synthesis

The decision was a result of the Reviewing Editor Leonard Maler and the peer reviewers coming together and discussing their recommendations until a consensus was reached. A fact-based synthesis statement explaining their decision and outlining what is needed to prepare a revision is listed below.

Both reviewers are in agreement on the importance of this Ms as it clarifies and corrects previous publications.

This Ms is therefore a very useful contribution to the field of cochlear afferents although its implications are relatively narrow and restricted to this field.

I have carefully read the revised Ms and the authors' rebuttal and find that all the reviewers concerns have been properly addressed.
